# Revisões Sistemáticas e Metanálises: Faróis na Tempestade de Informação da COVID-19

**DOI:** 10.36660/abc.20220442

**Published:** 2022-07-29

**Authors:** Henrique Turin Moreira, André Schmidt

**Affiliations:** 1 Universidade de São Paulo Faculdade de Medicina de Ribeirão Preto Hospital das Clínicas Ribeirão Preto SP Brasil Hospital das Clínicas da Faculdade de Medicina de Ribeirão Preto, Universidade de São Paulo, Ribeirão Preto, SP – Brasil

**Keywords:** COVID-19, Pandemia, Coronavírus, Síndrome Respiratória Aguda Grave, Revisão Sistemática, Metanálise

Com o reconhecimento da pandemia de COVID-19 como uma emergência de saúde pública, a comunidade científica internacional mobilizou-se na busca pelo conhecimento sobre a infecção pelo novo coronavírus denominado SARS-CoV-2. A necessidade de acelerar as pesquisas voltadas à redução da mortalidade e contenção do escalonamento da crise foi logo proclamada pela Organização Mundial da Saúde.^1^ Como resultado, observamos hoje uma extraordinária quantidade de dados sobre COVID-19, obtidos em um tempo muitíssimo curto. Uma busca pela base de dados Pubmed prontamente nos revela mais de 164.000 artigos científicos já publicados sobre a doença em menos de dois anos e meio, fenômeno sem precedentes na literatura médica. Colocando-se em perspectiva, essa profusão de publicações mostra-se numericamente maior do que aquelas identificadas pelo termo *myocardial infarction* nas últimas quatro décadas.

Embora esse formidável avanço científico tenha sido fundamental para o enfrentamento da pandemia, por outro lado, resultou em uma tempestade de informações com efeitos adversos bastante significativos. Profissionais da saúde enfrentaram dificuldades em buscar, interpretar e sobretudo sintetizar esse vertiginoso volume de evidências. Resultados conflitantes, muito comuns nos caminhos sinuosos da ciência, tornaram-se fontes frequentes de confusão e desentendimento.^2^ Em cenários turbulentos como esses, revisões sistemáticas e metanálises podem atuar como um farol, norteando rotas mais seguras a serem percorridas. Oferecem avaliação organizada e integrada de múltiplas fontes de dados, permitindo assim estimativas mais robustas e respostas mais confiáveis para os dilemas da prática clínica.

Nesse sentido, Barberato et al.,^3^ apresentam nesta edição dos Arquivos Brasileiros de Cardiologia uma revisão sistemática e metanálise sobre achados ecocardiográficos anormais em pacientes internados com COVID-19.^3^ A partir de 6.427 publicações inicialmente levantadas (já excluindo as duplicadas), os autores identificaram 38 artigos originais que preenchiam os critérios de seleção, todos publicados até junho de 2021. Como destaque, a disfunção sistólica do ventrículo esquerdo (VE) foi encontrada em um quarto dos casos pela ecocardiografia convencional, sendo identificada em até um terço dos pacientes pelo método de *speckle tracking.* Já a disfunção do ventrículo direito (VD) mostrou-se menos prevalente, presente em 17% dos indivíduos, enquanto hipertensão pulmonar e derrame pericárdico foram descritos em 23% e 17% dos casos respectivamente.

O acometimento cardíaco em pacientes com COVID-19 tem sido motivo de grande preocupação desde o início da pandemia.^4,5^ Evidências recentes mostram que a injúria miocárdica pela ação direta do vírus tem menor relevância do que as lesões indiretas pela inflamação sistêmica e hipercoagulobilidade nesses pacientes.^6,7^ A despeito da melhor compreensão desses mecanismos patogênicos, a prevalência do envolvimento miocárdico na COVID-19 permanece em debate. Por um lado, o diagnóstico de injúria miocárdica baseado exclusivamente na elevação de biomarcadores séricos, como a troponina, pode superestimar o número de casos.^8,9^ Métodos complementares como a ressonância magnética cardíaca e a biópsia endomiocárdicas, por outro lado, nem sempre estão disponíveis para confirmar o dano cardíaco.

Embora a revisão sistemática e metanálise de Barberato et al.,^3^ certamente contribua para a caracterização fenotípica das anormalidades cardíacas dos pacientes internados com COVID-19, os resultados apresentados levantam questões adicionais. O envolvimento cardíaco encontrado corresponderia a alterações pré-existentes ou seria decorrente da infecção pelo SARS-CoV-2? Para responder a essa pergunta, os autores reportam uma associação direta entre as anormalidades ecocardiográficas prévias e maiores proporções de disfunção sistólica do VE. Contudo, é importante ressaltar que apenas 8 dos 38 estudos (somente 9% de todos os pacientes agrupados) reportaram resultados de ecocardiogramas anteriores.

Adicionalmente, os resultados demonstrados são bastante relevantes também por outros aspectos. A heterogeneidade entre os estudos foi excessivamente elevada, aparentemente não explicada pela prevalência de doenças cardiovasculares pré-existentes ou pela proporção de pacientes em ventilação mecânica. Outros fatores poderiam explicar essa alarmante heterogeneidade, com destaque para pequenas amostras populacionais, diferenças entre os protocolos ecocardiográficos utilizados e singularidades demográficas e clínicas entre as populações de cada estudo.^10^ Além disso, a análise gráfica sugere fortemente a presença de viés de publicação de estudos documentando a disfunção sistólica do VD, com uma tendência de as investigações de menor tamanho amostral relatarem uma maior proporção desse achado.

Finalmente, hoje nos deparamos com um cenário significativamente diferente daquele avaliado pelos estudos até meados do ano passado. Expansão da cobertura vacinal, aparecimentos de variantes virais, reconhecimento de sintomas prolongados e do risco cardiovascular aumentado após o quadro agudo de COVID-19.^11–13^ Novas indagações emergem nesse contexto, especialmente sobre a prevalência de injúria cardíaca em quadros mais brandos, além do papel do envolvimento cardíaco nos casos de COVID-19 com sintomas prolongados e do valor prognóstico das lesões miocárdicas associadas à infecção.^14^

Nesses tempos de tormenta de informações, o estudo de Barberato et al.^3^ cumpre sua função de nos guiar em direção a conclusões mais confiáveis. Ademais, realça os contratempos e as armadilhas desse caminho apressado que a ciência percorre atualmente. Adiante, temos agora o desafio de compreender o fenótipo do envolvimento cardíaco em um novo cenário clínico e epidemiológico que já se anuncia.
