# Supplementary material for: Revisões Sistemáticas e Metanálises: Faróis na Tempestade de Informação da COVID-19
Source: Arq Bras Cardiol. 2022 Jul 29;119(2):280–1. [Article in Portuguese] doi: 10.36660/abc.20220442 (PMC9363051; doi:10.36660/abc.20220442)
Supplement: Translation

# Translation: Systematic Reviews and Meta-Analyses: Lighthouses in the Data Storm from the COVID-19 Pandemic

**Authors:** Henrique Turin Moreira, André Schmidt

**Affiliations:** 1 Ribeirão Preto SP Brazil Hospital das Clínicas da Faculdade de Medicina de Ribeirão Preto, Universidade de São Paulo, Ribeirão Preto, SP – Brazil

**Keywords:** COVID-19, Pandemics, Coronavirus, Severe Acute Respiratory Syndrome, Systematic Reviews, Meta-Analysis

Since recognizing the COVID-19 pandemic as a public health emergency, the global scientific community has driven efforts to understand the infection by the new coronavirus SARS-CoV-2. The World Health Organization soon proclaimed the need to fast-track research to reduce mortality and avoid crisis escalation.^1^ As a result, we now observe an extraordinary amount of data on COVID-19 obtained in a short time. Indeed, a search in the Pubmed database promptly reveals more than 164,000 papers on the disease in less than two and a half years, an unprecedented phenomenon in the medical literature. Putting this into perspective, this profusion of publications is numerically greater than papers identified by the term “myocardial infarction” in the last four decades.

Although this outstanding scientific advance has been crucial to fighting the pandemic, at the same time, it went along with a data storm with marked adverse effects. Health professionals had challenges searching, interpreting, and summarizing this dizzying volume of evidence. Conflicting results, typical of the twisted paths of science, were frequent causes of confusion and disagreement.^2^ In this setting, systematic reviews and meta-analyses can serve as lighthouses, guiding us to safer routes. They offer organized and integrated assessment of multiple data sources, thus allowing more robust estimates and reliable answers to clinical practice dilemmas.

For this purpose, Barberato et al.^3^ present in this edition of *Arquivos Brasileiros de Cardiologia* a systematic review and meta-analysis on abnormal echocardiographic findings in hospitalized patients with COVID-19.^3^ From 6,427 publications initially selected (already excluding duplicates), the authors identified 38 original articles that met the selection criteria, all published until June 2021. Noteworthy, left ventricular (LV) systolic dysfunction was found in a quarter of cases by conventional echocardiography and in up to a third of patients by the speckle tracking method. On the other hand, right ventricular (RV) dysfunction was less prevalent, present in 17% of individuals, while pulmonary hypertension and pericardial effusion were described in 23% and 17% of cases, respectively.

Cardiac involvement in patients with COVID-19 has been a concern since the pandemic’s beginning.^4,5^ Recent evidence shows that direct myocardial injury by the virus is less relevant than indirect lesions from systemic inflammation and hypercoagulability in these patients.^6,7^ Despite understanding these pathogenic mechanisms, the prevalence of myocardial involvement in COVID-19 remains debatable. Diagnosis of myocardial injury based exclusively on the elevation of serum biomarkers, such as troponin, may overestimate the number of cases.^8,9^ On the other hand, complementary methods such as cardiac magnetic resonance and endomyocardial biopsy are not always available to confirm cardiac damage.

Although the systematic review and meta-analysis by Barberato et al.^3^ certainly contribute to phenotyping the cardiac abnormalities in hospitalized patients with COVID-19, the findings raise other questions. For instance, would the cardiac damage correspond to pre-existing abnormalities or be a consequence of the SARS-CoV-2 infection? To answer this question, the authors reported a direct association between previous echocardiographic abnormalities and higher proportions of LV systolic dysfunction. However, it is essential to note that only 8 of the 38 studies (9% of all polled patients) described prior echocardiograms.

In addition, the meta-analysis’ findings underline other extremely relevant aspects. The heterogeneity of the studies was quite high, apparently not explained by the prevalence of pre-existing cardiovascular diseases or the proportion of patients on mechanical ventilation. This warning lack of homogeneity could be explained by other factors, emphasizing small sample sizes, differences between the echocardiographic protocols, and demographic and clinical singularities among the study populations.^10^ Furthermore, the graphical analysis strongly suggests the presence of publication bias in studies documenting RV systolic dysfunction, with a tendency for smaller sample size investigations to report a more significant proportion of this finding.

Finally, today we are faced with a quite different scenario from the one evaluated by the studies until the middle of last year: expansion of vaccine coverage, the appearance of viral variants, recognition of COVID-19 prolonged symptoms, and increased cardiovascular risk after the acute infection.^11–13^ Therefore, new questions emerged, especially regarding the prevalence of heart injury in milder conditions, the cardiac involvement’s role in long-term COVID-19, and the predictive value of the myocardial damage related to the infection.^14^

During the data storm from the COVID-19 pandemic, the study by Barberato et al.^3^ fulfills its aim of guiding us towards more reliable conclusions. Furthermore, and equally relevant, it highlights the shortcomings and pitfalls of a rushing science. Further ahead, we now have the challenge of phenotyping the cardiac involvement in a new and already announced clinical and epidemiological scenario.
